# Supplementary material for: DCC regulates astroglial development essential for telencephalic morphogenesis and corpus callosum formation
Source: eLife. 2021 Apr 19;10:e61769. doi: 10.7554/eLife.61769 (PMC8116049; doi:10.7554/eLife.61769)
Supplement: Supplementary file 1. [file elife-61769-supp1.docx]

Supplementary File 1: Statistics

| **Figure** | **Data compared** | **Number of samples** | **Statistical test** | **p value** |
| --- | --- | --- | --- | --- |
| 1B | Dcc knockout IHF length | n = 5 wt, n = 7 exp | Mann-Whitney test | 0.0012 |
| 1B | DCCK IHF length | n = 6 both conditions | Mann-Whitney test | 0.0022 |
| 1B | Ntn1-lacZ IHF length | n = 11 wt, n = 6 exp | Mann-Whitney test | 0.0002 |
| 1B | Dcc knockout vs. DCCK IHF length | n = 7 Dcc knockout, n = 6 DCCK | Mann-Whitney test | 0.9452 |
| 1B | Dcc knockout vs. Ntn1-lacZ IHF length | n = 7 Dcc knockout, n = 6 Ntn1-lacZ | Mann-Whitney test | 0.9452 |
| 1B | DCCK vs. Ntn1-lacZ IHF length | n = 6 both conditions | Mann-Whitney test | 0.5887 |
| 3G | E14 DCCK GLAST FI whole ROI | n = 5 exp, n = 6 wt | Mann-Whitney test | 0.1255 |
| 3G | E14 DCCK GLAST FI 0-50µm | n = 5 exp, n = 6 wt | Mann-Whitney test | 0.0043 |
| 3G | E14 DCCK GLAST FI 50-150µm | n = 5 exp, n = 6 wt | Mann-Whitney test | 0.7922 |
| 3G | E14 DCCK GLAST FI 150-200µm | n = 5 exp, n = 6 wt | Mann-Whitney test | 0.0303 |
| 3H | E15 DCCK GLAST FI whole ROI | n = 6 exp, n = 7 wt | Mann-Whitney test | 0.014 |
| 3H | E15 DCCK GLAST FI 0-50µm | n = 6 exp, n = 7 wt | Mann-Whitney test | 0.366 |
| 3H | E15 DCCK GLAST FI 50-150µm | n = 6 exp, n = 7 wt | Mann-Whitney test | 0.0082 |
| 3H | E15 DCCK GLAST FI 150-200µm | n = 6 exp, n = 7 wt | Mann-Whitney test | 0.0221 |
| 3I | E16 DCCK GLAST FI whole ROI | n = 6 exp, n = 8 wt | Mann-Whitney test | 0.9497 |
| 3I | E16 DCCK GLAST FI 0-50µm | n = 6 exp, n = 8 wt | Mann-Whitney test | 0.2824 |
| 3I | E16 DCCK GLAST FI 50-150µm | n = 6 exp, n = 8 wt | Mann-Whitney test | 0.4136 |
| 3I | E16 DCCK GLAST FI 150-200µm | n = 6 exp, n = 8 wt | Mann-Whitney test | 0.9497 |
| 3J | E14 DCCK NESTIN distribution | n = 6 both conditions | Mann-Whitney test | 0.0022 |
| 3J | E15 DCCK NESTIN distribution | n = 7 exp, n =8 wt | Mann-Whitney test | 0.0022 |
| 3J | E16 DCCK NESTIN distribution | n = 6 exp, n = 7 wt | Mann-Whitney test | 0.1014 |
| 3J | E14 vs. E15 wt NESTIN distribution | n = 6 E14, n = 8 E15 | Mann-Whitney test | 0.1419 |
| 3J | E14 vs. E15 DCCK NESTIN distribution | n = 6 E14, n = 7 E15 | Mann-Whitney test | 0.0047 |
| 3J | E15 vs. E16 wt NESTIN distribution | n = 8 E15, n = 7 E16 | Mann-Whitney test | 0.0059 |
| 3J | E15 vs. E16 DCCK NESTIN distribution | n = 7 E15, n = 6 E16 | Mann-Whitney test | 0.0012 |
| 3J | E14 DCCK GLAST distribution | n = 6 both conditions | Mann-Whitney test | 0.0152 |
| 3J | E15 DCCK GLAST distribution | n = 7 exp, n =8 wt | Mann-Whitney test | 0.0012 |
| 3J | E16 DCCK GLAST distribution | n = 6 exp, n = 7 wt | Mann-Whitney test | 0.2949 |
| 3J | E14 vs. E15 wt GLAST distribution | n = 6 E14, n = 8 E15 | Mann-Whitney test | 0.0127 |
| 3J | E14 vs. E15 DCCK GLAST distribution | n = 6 E14, n = 7 E15 | Mann-Whitney test | 0.2343 |
| 3J | E15 vs. E16 wt GLAST distribution | n = 8 E15, n = 7 E16 | Mann-Whitney test | 0.0541 |
| 3J | E15 vs. E16 DCCK GLAST distribution | n = 7 E15, n = 6 E16 | Mann-Whitney test | 0.0023 |
| 4B | E14 SOX9 MZG DCCK 0-50µm | n = 8 exp, n = 12 wt | Two-way ANOVA with Sidak's multiple comparisons test | >0.9999 |
| 4B | E14 SOX9 MZG DCCK 50-100µm | n = 8 exp, n = 12 wt | Two-way ANOVA with Sidak's multiple comparisons test | 0.8565 |
| 4B | E14 SOX9 MZG DCCK 100-150µm | n = 8 exp, n = 12 wt | Two-way ANOVA with Sidak's multiple comparisons test | 0.9374 |
| 4B | E14 SOX9 MZG DCCK 150-200µm | n = 8 exp, n = 12 wt | Two-way ANOVA with Sidak's multiple comparisons test | >0.9999 |
| 4B | E14 SOX9 MZG DCCK 200-250µm | n = 8 exp, n = 12 wt | Two-way ANOVA with Sidak's multiple comparisons test | 0.9996 |
| 4D | E15 SOX9 MZG DCCK 0-50µm | n = 11 exp, n = 9 wt | Two-way ANOVA with Sidak's multiple comparisons test | 0.9275 |
| 4D | E15 SOX9 MZG DCCK 50-100µm | n = 11 exp, n = 9 wt | Two-way ANOVA with Sidak's multiple comparisons test | 0.9934 |
| 4D | E15 SOX9 MZG DCCK 100-150µm | n = 11 exp, n = 9 wt | Two-way ANOVA with Sidak's multiple comparisons test | 0.8976 |
| 4D | E15 SOX9 MZG DCCK 150-200µm | n = 11 exp, n = 9 wt | Two-way ANOVA with Sidak's multiple comparisons test | 0.4158 |
| 4D | E15 SOX9 MZG DCCK 200-250µm | n = 11 exp, n = 9 wt | Two-way ANOVA with Sidak's multiple comparisons test | 0.0042 |
| 4D | E15 SOX9 MZG DCCK 250-300µm | n = 11 exp, n = 9 wt | Two-way ANOVA with Sidak's multiple comparisons test | 0.7722 |
| 4F | E16 SOX9 MZG DCCK 0-50µm | n = 6 exp, n = 7 wt | Two-way ANOVA with Sidak's multiple comparisons test | 0.026 |
| 4F | E16 SOX9 MZG DCCK 50-100µm | n = 6 exp, n = 7 wt | Two-way ANOVA with Sidak's multiple comparisons test | <0.0001 |
| 4F | E16 SOX9 MZG DCCK 100-150µm | n = 6 exp, n = 7 wt | Two-way ANOVA with Sidak's multiple comparisons test | <0.0001 |
| 4F | E16 SOX9 MZG DCCK 150-200µm | n = 6 exp, n = 7 wt | Two-way ANOVA with Sidak's multiple comparisons test | <0.0001 |
| 4F | E16 SOX9 MZG DCCK 200-250µm | n = 6 exp, n = 7 wt | Two-way ANOVA with Sidak's multiple comparisons test | 0.0012 |
| 4F | E16 SOX9 MZG DCCK 250-300µm | n = 6 exp, n = 7 wt | Two-way ANOVA with Sidak's multiple comparisons test | 0.73 |
| 4F | E16 SOX9 MZG DCCK 300-350µm | n = 6 exp, n = 7 wt | Two-way ANOVA with Sidak's multiple comparisons test | 0.5323 |
| 4F | E16 SOX9 MZG DCCK 350-400µm | n = 6 exp, n = 7 wt | Two-way ANOVA with Sidak's multiple comparisons test | 0.9664 |
| 4G | E14 SOX9 MZG DCCK TOTAL | n = 8 exp, n =12 wt | Two-way unpaired Student's t test | 0.6584 |
| 4G | E15 SOX9 MZG DCCK TOTAL | n = 11 exp, n = 9 wt | Two-way unpaired Student's t test | 0.0214 |
| 4G | E16 SOX9 MZG DCCK TOTAL | n = 6 exp, n = 7 wt | Mann-Whitney test | 0.0017 |
| 4G | E14 wt vs. E15 wt SOX9 MZG TOTAL | n = 12 E14, n = 9 E15 | Two-way unpaired Student's t test | 0.0023 |
| 4G | E14 DCCK vs. E15 DCCK SOX9 MZG TOTAL | n = 8 E14, n = 11 E15 | Two-way unpaired Student's t test | 0.5572 |
| 4G | E15 wt vs. E16 wt SOX9 MZG TOTAL | n = 9 E15, n = 7 E16 | Mann-Whitney test | 0.2991 |
| 4G | E15 DCCK vs. E16 DCCK SOX9 MZG TOTAL | n = 11 E15, n = 6 E16 | Mann-Whitney test | 0.0002 |
| 5B | E17 Mean normalised GFAP FI *Ntn1-lacZ* | n = 6 exp; n = 18 wt | Kruskal-Wallis test with post-hoc Dunn’s multiple comparison test. | 0.4709 |
| 5B | E17 Mean normalised GFAP FI *Dcc* knockout | n = 7 exp; n = 18 wt | Kruskal-Wallis test with post-hoc Dunn’s multiple comparison test. | > 0.9999 |
| 5B | E17 Mean normalised GFAP FI *Dcc^kanga^* | n = 6 exp; n = 18 wt | Kruskal-Wallis test with post-hoc Dunn’s multiple comparison test. | > 0.9999 |
| 5C | E17 Ratio mean normalised GFAP FI rostral over caudal bin *Ntn1-lacZ* | n = 6 exp; n = 18 wt | Kruskal-Wallis test with post-hoc Dunn’s multiple comparison test. | 0.0009 |
| 5C | E17 Ratio mean normalised GFAP FI rostral over caudal bin *Dcc* knockout | n = 7 exp; n = 18 wt | Kruskal-Wallis test with post-hoc Dunn’s multiple comparison test. | 0.0004 |
| 5C | E17 Ratio mean normalised GFAP FI rostral over caudal bin *Dcc^kanga^* | n = 6 exp; n = 18 wt | Kruskal-Wallis test with post-hoc Dunn’s multiple comparison test. | 0.0008 |
| 6E | Ratio IHF length P0 *Dcc* cKO mice ventral | n = 12 exp; n = 6 control | Mann-Whitney test | 0.0004 |
| 6E | Ratio IHF length P0 *Dcc* cKO mice middle | n = 12 exp; n = 6 control | Mann-Whitney test | <0.0001 |
| 6E | Ratio IHF length P0 *Dcc* cKO mice dorsal | n = 12 exp; n = 6 control | Mann-Whitney test | 0.0011 |
| 6F | Ratio CC length P0 *Dcc* cKO mice ventral | n = 12 exp; n = 6 control | Mann-Whitney test | 0.0080 |
| 6F | Ratio CC length P0 *Dcc* cKO mice middle | n = 12 exp; n = 6 control | Mann-Whitney test | 0.1861 |
| 6F | Ratio CC length P0 *Dcc* cKO mice dorsal | n = 12 exp; n = 6 control | Mann-Whitney test | 0.078 |
| 6G | Ratio CC depth P0 *Dcc* cKO mice ventral | n = 12 exp; n = 6 control | Mann-Whitney test | 0.0476 |
| 6G | Ratio CC depth P0 *Dcc* cKO mice middle | n = 12 exp; n = 6 control | Mann-Whitney test | 0.0006 |
| 6G | Ratio CC depth P0 *Dcc* cKO mice dorsal | n = 12 exp; n = 6 control | Mann-Whitney test | 0.0190 |
| 6H | Ratio HC length P0 *Dcc* cKO mice ventral | n = 12 exp; n = 6 control | Mann-Whitney test | 0.0047 |
| 6H | Ratio HC length P0 *Dcc* cKO mice middle | n = 12 exp; n = 6 control | Mann-Whitney test | 0.6495 |
| 6H | Ratio HC length P0 *Dcc* cKO mice dorsal | n = 12 exp; n = 6 control | Mann-Whitney test | 0.7559 |
| 6I | DCC expression P0 *Dcc* cKO mice cingulate cortex | n = 15 exp; n = 6 control | Mann-Whitney test | 0.0016 |
| 6I | DCC expression P0 *Dcc* cKO mice intermediate zone | n = 15 exp; n = 6 control | Mann-Whitney test | <0.0001 |
| 6J | Pearson r correlation between ratio CC length versus IHF length in middle *Dcc* cKO P0 sections | n = 8 control | Pearson correlation | 0.2765 |
| 6J | Pearson r correlation between ratio CC length versus IHF length in middle *Dcc* cKO P0 sections | n = 12 exp | Pearson correlation | 0.0002 |
| 6K | Pearson r correlation between ratio HC length versus IHF length in middle *Dcc* cKO P0 sections | n = 8 control | Pearson correlation | 0.3491 |
| 6K | Pearson r correlation between ratio HC length versus IHF length in middle *Dcc* cKO P0 sections | n = 12 exp | Pearson correlation | 0.0009 |
| 6O | FI of DCC expression E15 *Dcc* cKO mice cingulate cortex | n = 6 exp, n = 5 control | Mann-Whitney test | 0.0173 |
| 6O | FI of DCC expression E15 *Dcc* cKO mice MZG | n = 6 exp, n = 5 control | Mann-Whitney test | 0.6623 |
| 6P | FI of GAP43 expression E15 *Dcc* cKO mice IHF surface | n = 6 exp, n = 5 control | Mann-Whitney test | 0.2468 |
| 7D | U251 CA myr-TDT vs. DCC:TDT vehicle | n = 72 wt DCC, n = 67 control | Kruskal-Wallis test with Dunn's multiple comparisons test | <0.0001 |
| 7D | U251 CA DCC:TDT vs. DCCK:TDT vehicle | n = 77 DCCK, n = 72 wt DCC | Kruskal-Wallis test with Dunn's multiple comparisons test | <0.0001 |
| 7D | U251 CA myr-TDT vs. DCCK:TDT vehicle | n = 77 DCCK, n = 67 control | Kruskal-Wallis test with Dunn's multiple comparisons test | >0.9999 |
| 7D | U251 CA myr-TDT vs. DCC:TDT NTN1 | n = 82 wt DCC, n = 65 control | Kruskal-Wallis test with Dunn's multiple comparisons test | 0.0015 |
| 7D | U251 CA DCC:TDT vs. DCCK:TDT NTN1 | n = 71 DCCK, n = 82 wt DCC | Kruskal-Wallis test with Dunn's multiple comparisons test | 0.0027 |
| 7D | U251 CA myr-TDT vs. DCCK:TDT NTN1 | n = 71 DCCK, n = 65 control | Kruskal-Wallis test with Dunn's multiple comparisons test | >0.9999 |
| 7D | U251 CA DCC:TDT vehicle vs. DCC:TDT NTN1 | n = 72 vehicle, n = 82 NTN1 | Kruskal-Wallis test with Dunn's multiple comparisons test | >0.9999 |
| 7E | U251 CP myr-TDT vs. DCC:TDT vehicle | n = 72 wt DCC, n = 68 control | Kruskal-Wallis test with Dunn's multiple comparisons test | <0.0001 |
| 7E | U251 CP DCC:TDT vs. DCCK:TDT vehicle | n = 77 DCCK, n = 72 wt DCC | Kruskal-Wallis test with Dunn's multiple comparisons test | <0.0001 |
| 7E | U251 CP myr-TDT vs. DCCK:TDT vehicle | n = 77 DCCK, n = 68 control | Kruskal-Wallis test with Dunn's multiple comparisons test | >0.9999 |
| 7E | U251 CP myr-TDT vs. DCC:TDT NTN1 | n = 82 wt DCC, n = 67 control | Kruskal-Wallis test with Dunn's multiple comparisons test | <0.0001 |
| 7E | U251 CP DCC:TDT vs. DCCK:TDT NTN1 | n = 71 DCCK, n = 82 wt DCC | Kruskal-Wallis test with Dunn's multiple comparisons test | 0.0158 |
| 7E | U251 CP myr-TDT vs. DCCK:TDT NTN1 | n = 71 DCCK, n = 67 control | Kruskal-Wallis test with Dunn's multiple comparisons test | >0.9999 |
| 7E | U251 CP DCC:TDT vehicle vs. DCC:TDT NTN1 | n = 72 vehicle, n = 82 NTN1 | Kruskal-Wallis test with Dunn's multiple comparisons test | >0.9999 |
| 7F | U251 CC myr-TDT vs. DCC:TDT vehicle | n = 72 wt DCC, n = 68 control | Kruskal-Wallis test with Dunn's multiple comparisons test | 0.0006 |
| 7F | U251 CC DCC:TDT vs. DCCK:TDT vehicle | n = 77 DCCK, n = 72 wt DCC | Kruskal-Wallis test with Dunn's multiple comparisons test | <0.0001 |
| 7F | U251 CC myr-TDT vs. DCCK:TDT vehicle | n = 77 DCCK, n = 68 control | Kruskal-Wallis test with Dunn's multiple comparisons test | >0.9999 |
| 7F | U251 CC myr-TDT vs. DCC:TDT NTN1 | n = 82 wt DCC, n = 67 control | Kruskal-Wallis test with Dunn's multiple comparisons test | 0.0052 |
| 7F | U251 CC DCC:TDT vs. DCCK:TDT NTN1 | n = 71 DCCK, n = 82 wt DCC | Kruskal-Wallis test with Dunn's multiple comparisons test | >0.9999 |
| 7F | U251 CC myr-TDT vs. DCCK:TDT NTN1 | n = 71 DCCK, n = 67 control | Kruskal-Wallis test with Dunn's multiple comparisons test | >0.9999 |
| 7F | U251 CC DCC:TDT vehicle vs. DCC:TDT NTN1 | n = 72 vehicle, n = 82 NTN1 | Kruskal-Wallis test with Dunn's multiple comparisons test | >0.9999 |
| 7I | N2A CA myr-TDT vs. DCC:TDT vehicle | n = 55 wt DCC, n = 90 control | Kruskal-Wallis test with Dunn's multiple comparisons test | 0.0003 |
| 8E | N2A CA myr-TDT vs. DCC:TDT NTN1 | n = 191 wt DCC, n = 100 control | Kruskal-Wallis test with Dunn's multiple comparisons test | 0.0005 |
| 8E | N2A CA myr-TDT vs. DCC(M743L):TDT NTN1 | n = 77 exp, n = 100 control | Kruskal-Wallis test with Dunn's multiple comparisons test | >0.9999 |
| 8E | N2A CA myr-TDT vs. DCC(V754M):TDT NTN1 | n = 95 exp, n = 100 control | Kruskal-Wallis test with Dunn's multiple comparisons test | >0.9999 |
| 8E | N2A CA myr-TDT vs. DCC(A893T):TDT NTN1 | n = 71 exp, n = 100 control | Kruskal-Wallis test with Dunn's multiple comparisons test | >0.9999 |
| 8E | N2A CA myr-TDT vs. DCC(V793G):TDT NTN1 | n = 86 exp, n = 100 control | Kruskal-Wallis test with Dunn's multiple comparisons test | >0.9999 |
| 8E | N2A CA myr-TDT vs. DCC(G805E):TDT NTN1 | n = 80exp, n = 100 control | Kruskal-Wallis test with Dunn's multiple comparisons test | 0.1063 |
| 8E | N2A CA myr-TDT vs. DCC(M1217V;A1250T):TDT NTN1 | n = 75 exp, n = 100 control | Kruskal-Wallis test with Dunn's multiple comparisons test | >0.9999 |
| 8E | N2A CA myr-TDT vs. DCC(V848R):TDT NTN1 | n = 92 exp, n = 100 control | Kruskal-Wallis test with Dunn's multiple comparisons test | 0.7138 |
| 8E | N2A CA myr-TDT vs. DCC(H857A):TDT NTN1 | n = 77 exp, n = 100 control | Kruskal-Wallis test with Dunn's multiple comparisons test | >0.9999 |
| 1-S1 | Adult DCCK IHF length | n = 4 wt, n = 6 exp | Mann-Whitney test | 0.003 |
| 3-S1D | Fl of β-dystroglycan along IHF pial surface in DCCK E15 mice | n = 5 DCCK, n = 5 wt | Mann-Whitney test | 0.4206 |
| 3-S1F | FI of APC along IHF pial surface in DCCK E15 mice | n = 5 DCCK, n = 5 wt | Mann-Whitney test | 0.0159 |
| 3-S1F | FI of N-CADHERIN along IHF pial surface in DCCK E15 mice | n = 6 DCCK, n = 5 wt | Mann-Whitney test | 0.5368 |
| 3-S1F | FI of β-catenin along IHF pial surface in DCCK E15 mice | n = 6 DCCK, n = 5 wt | Mann-Whitney test | 0.9307 |
| 3-S1G | FI of NESTIN along IHF pial surface in DCCK E15 mice | n = 6 DCCK, n = 5 wt | Mann-Whitney test | 0.6623 |
| 4-C | E12-E13 EdU+/Ki67+ MZG DCCK | n= 6 DCCK, n = 7 wt | Mann-Whitney test | 0.4219 |
| 4-S1C | E13-E14 EdU+/Ki67+ MZG DCCK | n= 8 DCCK, n = 6 wt | Mann-Whitney test | 0.1512 |
| 4-S1C | E14-E15 EdU+/Ki67+ MZG DCCK | n= 8 DCCK, n = 6wt | Mann-Whitney test | 0.0939 |
| 4-S1D | E12-E13 EdU+/Ki67- MZG DCCK | n= 6 DCCK, n = 7 wt | Mann-Whitney test | 0.6503 |
| 4-S1D | E13-E14 EdU+/Ki67- MZG DCCK | n= 8 DCCK, n = 6 wt | Mann-Whitney test | 0.1419 |
| 4-S1D | E14-E15 EdU+/Ki67- MZG DCCK | n= 8 DCCK, n = 6 wt | Mann-Whitney test | 0.8019 |
| 4-S1F | E13 Cleaved-CASPASE3+ cells DCCK | n=4 DCCK, n = 5 wt | Mann-Whitney test | 0.9524 |
| 4-S1F | E14 Cleaved-CASPASE3+ cells DCCK | n = 7 both conditions | Mann-Whitney test | 0.0816 |
| 4-S1F | E15 Cleaved-CASPASE3+ cells DCCK | n = 8 both conditions | Mann-Whitney test | 0.5611 |
| 4-S1I | E16 SOX9 IGGDCCK TOTAL | n = 5 exp, n = 6 wt | Mann-Whitney test | 0.0043 |
| 5-S1A | E15 DCCK MZG GFAP FI | n = 6 exp, n = 6 wt | Mann-Whitney test | 0.5887 |
| 5-S1D | E15 DCCK Fgf8 mRNA chromogenic intensity ratio | n = 6 exp, n = 3 wt | Mann-Whitney test | >0.9999 |
| 5-S1D | E15 DCCK Mmp-2 mRNA chromogenic intensity ratio | n = 5 exp, n = 4 wt | Mann-Whitney test | 0.1111 |
| 5-S1G | E14 DCCK NFIA MZG | n = 8 exp, n = 6 wt | Mann-Whitney test | 0.8691 |
| 5-S1G | E14 DCCK NFIB MZG | n = 9 exp, n = 5 wt | Mann-Whitney test | 0.8227 |
| 6-S1C | Ratio DCC expression EP/non EP hemisphere wildtype mice *Dcc-CRISPR* | n = 3 exp, n = 5 wt | Mann-Whitney test | >0.9999 |
| 6-S1C | Ratio DCC expression EP/non EP hemisphere DCCK heterozygous mice *Dcc-CRISPR* | n = 9 exp, n =9 wt | Unpaired t test | 0.5412 |
| 6-S1C | Ratio DCC expression EP/non EP hemisphere DCCK heterozygous mice *Dcc-shRNA* | n = 4 exp, n =9 wt | Mann-Whitney test | 0.0196 |
| 6-S1D | Spearman r correlation between ratio CC length versus IHF length in ventral *Dcc* cKO P0 sections | n = 6 control | Spearman correlation | 0.9194 |
| 6-S1D | Spearman r correlation between ratio CC length versus IHF length in ventral *Dcc* cKO P0 sections | n = 12 exp | Spearman correlation | 0.0455 |
| 6-S1E | Spearman r correlation between ratio HC length versus IHF length in ventral *Dcc* cKO P0 sections | n = 6 control | Spearman correlation | >0.9999 |
| 6-S1E | Spearman r correlation between ratio HC length versus IHF length in ventral *Dcc* cKO P0 sections | n = 12 exp | Spearman correlation | 0.0055 |
| 6-S1F | Spearman r correlation between ratio CC length versus IHF length in dorsal *Dcc* cKO P0 sections | n = 7 control | Spearman correlation | 0.0238 |
| 6-S1F | Spearman r correlation between ratio CC length versus IHF length in dorsal *Dcc* cKO P0 sections | n = 12 exp | Spearman correlation | 0.0002 |
| 6-S1G | Spearman r correlation between ratio HC length versus IHF length in dorsal *Dcc* cKO P0 sections | n = 7 control | Spearman correlation | 0.1095 |
| 6-S1G | Spearman r correlation between ratio HC length versus IHF length in dorsal *Dcc* cKO P0 sections | n = 11 exp | Spearman correlation | 0.0188 |
| 6-S1I | FI of GLAST along IHF EMX1 *Dcc* cKO total | n = 6 exp, n = 5 control | Mann-Whitney test | >0.9999 |
| 6-S1I | FI of GLAST along IHF EMX1 *Dcc* cKO 0-50µm | n = 6 exp, n = 5 control | Mann-Whitney test | >0.9999 |
| 6-S1I | FI of GLAST along IHF EMX1 *Dcc* cKO 50-100µm | n = 6 exp, n = 5 control | Mann-Whitney test | 0.7922 |
| 6-S1I | FI of GLAST along IHF EMX1 *Dcc* cKO 100-150µm | n = 6 exp, n = 5 control | Mann-Whitney test | 0.7922 |
| 6-S1I | FI of GLAST along IHF EMX1 *Dcc* cKO 150-200µm | n = 6 exp, n = 5 control | Mann-Whitney test | 0.7922 |
| 7-8B | U251 CA TDT vs. DCC:TDT | n = 49 wt DCC, n = 57 control | Kruskal-Wallis test with Dunn's multiple comparisons test | <0.0001 |
| 7-S1B | U251 CA DCC:TDT vs. DCCK:TDT | n = 49 wt DCC, n = 59 DCCK | Kruskal-Wallis test with Dunn's multiple comparisons test | 0.0082 |
| 7-S1B | U251 CA TDT vs. DCCK:TDT | n = 49 wt DCC, n = 59 DCCK | Kruskal-Wallis test with Dunn's multiple comparisons test | 0.0145 |
| 7-S1G | N2A CP myr-TDT vs. DCC:TDT vehicle | n = 55 wt DCC, n = 90 control | Kruskal-Wallis test with Dunn's multiple comparisons test | <0.0001 |
| 7-S1G | N2A CP myr-TDT vs. DCC:TDT NTN1 | n= 191 wt DCC, n = 100 control | Kruskal-Wallis test with Dunn's multiple comparisons test | 0.0014 |
| 7-S1G | N2A CP myr-TDT vs. DCC(M743L):TDT NTN1 | n = 77 exp, n = 100 control | Kruskal-Wallis test with Dunn's multiple comparisons test | >0.9999 |
| 7-S1G | N2A CP myr-TDT vs. DCC(V754M):TDT NTN1 | n = 95 exp, n = 100 control | Kruskal-Wallis test with Dunn's multiple comparisons test | >0.9999 |
| 7-S1G | N2A CP myr-TDT vs. DCC(A893T):TDT NTN1 | n = 71 exp, n = 100 control | Kruskal-Wallis test with Dunn's multiple comparisons test | >0.9999 |
| 7-S1G | N2A CP myr-TDT vs. DCC(V793G):TDT NTN1 | n = 86 exp, n = 100 control | Kruskal-Wallis test with Dunn's multiple comparisons test | >0.9999 |
| 7-S1G | N2A CP myr-TDT vs. DCC(G805E):TDT NTN1 | n = 80exp, n = 100 control | Kruskal-Wallis test with Dunn's multiple comparisons test | >0.9999 |
| 7-S1G | N2A CP myr-TDT vs. DCC(M1217V;A1250T):TDT NTN1 | n = 75 exp, n = 100 control | Kruskal-Wallis test with Dunn's multiple comparisons test | >0.9999 |
| 7-S1G | N2A CP myr-TDT vs. DCC(V848R):TDT NTN1 | n = 92 exp, n = 100 control | Kruskal-Wallis test with Dunn's multiple comparisons test | >0.9999 |
| 7-S1G | N2A CP myr-TDT vs. DCC(H857A):TDT NTN1 | n = 77 exp, n = 100 control | Kruskal-Wallis test with Dunn's multiple comparisons test | >0.9999 |
| 7-S1G | N2A CP DCC:TDT vehicle vs. NTN1 | n= 191 wt DCC vehicle, n = 100 NTN1 | Kruskal-Wallis test with Dunn's multiple comparisons test | >0.9999 |
| 7-S1H | N2A CC myr-TDT vs. DCC:TDT vehicle | n = 55 wt DCC, n = 90 control | Kruskal-Wallis test with Dunn's multiple comparisons test | 0.0001 |
| 7-S1H | N2A CC myr-TDT vs. DCC:TDT NTN1 | n= 191 wt DCC, n = 100 control | Kruskal-Wallis test with Dunn's multiple comparisons test | 0.3311 |
| 7-S1H | N2A CC myr-TDT vs. DCC(M743L):TDT NTN1 | n = 77 exp, n = 100 control | Kruskal-Wallis test with Dunn's multiple comparisons test | >0.9999 |
| 7-S1H | N2A CC myr-TDT vs. DCC(V754M):TDT NTN1 | n = 95 exp, n = 100 control | Kruskal-Wallis test with Dunn's multiple comparisons test | >0.9999 |
| 7-S1H | N2A CC myr-TDT vs. DCC(A893T):TDT NTN1 | n = 71 exp, n = 100 control | Kruskal-Wallis test with Dunn's multiple comparisons test | 0.5619 |
| 7-S1H | N2A CC myr-TDT vs. DCC(V793G):TDT NTN1 | n = 86 exp, n = 100 control | Kruskal-Wallis test with Dunn's multiple comparisons test | >0.9999 |
| 7-S1H | N2A CC myr-TDT vs. DCC(G805E):TDT NTN1 | n = 80exp, n = 100 control | Kruskal-Wallis test with Dunn's multiple comparisons test | >0.9999 |
| 7-S1H | N2A CC myr-TDT vs. DCC(M1217V;A1250T):TDT NTN1 | n = 75 exp, n = 100 control | Kruskal-Wallis test with Dunn's multiple comparisons test | >0.9999 |
| 7-S1H | N2A CC myr-TDT vs. DCC(V848R):TDT NTN1 | n = 92 exp, n = 100 control | Kruskal-Wallis test with Dunn's multiple comparisons test | >0.9999 |
| 7-S1H | N2A CC myr-TDT vs. DCC(H857A):TDT NTN1 | n = 77 exp, n = 100 control | Kruskal-Wallis test with Dunn's multiple comparisons test | >0.9999 |
| 7-S1H | N2A CC DCC:TDT vehicle vs. NTN1 | n= 191 wt DCC vehicle, n = 100 NTN1 | Kruskal-Wallis test with Dunn's multiple comparisons test | >0.9999 |
| 7-S1I | U251 CC3 DCC:TDT vs. MYR-TDT | n = 3 per group | Mann-Whitney test | 0.7 |
| 7-S1I | N2A CC3 DCC:TDT vs. MYR-TDT | n = 3 per group | Mann-Whitney test | >0.9999 |

CA = cell area, CP = cell perimeter, CC3 = cleaved-caspase 3DCCK = DCCKanga, E = embryonic day, EP = electroporated, exp = experimental, FI = fluorescence intensity, IGG = indusium griseum glia, MZG = midline zipper glia, P = postnatal day, ROI = region of interest, TDT = TDTOMATO, vs. = versus, wt = wildtype.
